# *Scutellaria baicalensis* Georgi: A Promising Source of Bioactive Molecules for Kidney Disease Therapy

**DOI:** 10.3390/biom16010064

**Published:** 2025-12-31

**Authors:** Xia Yang, Fang Dou, Lang Hai, Yating Xiao, Jie Cui, Yangyang Cai, Rui Wang, Kai Ji, Yalong Feng, Hua Chen

**Affiliations:** 1 Key Laboratory of Protection, Development and Utilization of Medicinal Resources in Liupanshan Area, Ministry of Education, School of Pharmacy, Ningxia Medical University, No. 1160 Shengli Street, Yinchuan 750004, China; 2 School of Medical Care, Sichuan Vocational and Technical College, No. 1 Xuefu North Road, Suining 629000, China; 3 School of Chemistry and Chemical Engineering, Xianyang Normal University, Xianyang 712000, China

**Keywords:** *Scutellaria baicalensis* Georgi, radix scutellariae, flavonoids, acute kidney injury, chronic kidney disease

## Abstract

The incidence of kidney diseases has been increasing due to changes in modern lifestyles and the ecological environment. The progression of kidney disease is characterized by ongoing renal damage and a gradual decline in renal function, ultimately leading to end-stage renal disease. The limitations of present medications have brought many disadvantages to patients. Consequently, identifying bioactive molecules has emerged as a critical strategy in the development of novel therapies for kidney diseases, particularly those derived from natural medicinal resources. This review presents a comprehensive analysis of renoprotective effects and underlying mechanisms of the medicinal plant *Scutellaria baicalensis* Georgi based on evidence retrieved from multiple databases, including Web of Science, PubMed, and CNKI. Flavonoids from *S. baicalensis* have been demonstrated to have good renoprotective properties by mitigating inflammation and oxidative stress, inhibiting cell apoptosis, reducing renal fibrosis, etc. Baicalein, wogonin, baicalin, and wogonoside are considered as the main bioactive components of the renoprotective effect of *S. baicalensis*. Further research on candidate molecules derived from *S. baicalensis* represents a promising strategy for the development of novel therapeutic agents targeting kidney diseases.

## 1. Introduction

Kidney disease has emerged as a significant global public health challenge, clinically classified into two primary categories: acute kidney injury (AKI) and chronic kidney disease (CKD). According to the World Health Organization’s report, AKI exhibits an annual incidence exceeding 2000 cases per million population, with severe instances demonstrating mortality rates ranging from 50% to 80% [1]. Concurrently, CKD is characterized by a progressive decline in renal function and affects an estimated 850 million individuals worldwide, representing approximately 11% of the global population [2]. The epidemiological burden of renal pathologies is on the rise due to demographic aging, increasing prevalence of metabolic disorders—including diabetes mellitus and obesity—and escalating comorbidities associated with cardiovascular diseases. These converging health challenges highlight the urgency for enhanced preventive strategies and optimized disease management protocols.

The current clinical management of renal diseases primarily relies on pharmacotherapy, nutritional support therapy (e.g., low-protein diets and restricting salt, potassium, and phosphorus intake), renal replacement therapy, adjunctive treatment with traditional Chinese medicine, and lifestyle interventions [3,4,5,6,7]. Among these modalities, pharmacotherapy remains the cornerstone of treatment, which includes the use of renin–angiotensin–aldosterone system inhibitors to control hypertension and proteinuria, immunosuppressants for autoimmune nephropathies, and glucocorticoids to modulate immune responses. While these approaches demonstrate partial efficacy in slowing disease progression, they are accompanied by significant limitations and adverse effects. Firstly, a lack of specific targeted therapies results in supportive treatments being the primary strategy. Secondly, most interventions focus predominantly on delaying disease progression rather than reversing it. Thirdly, many treatments are associated with adverse reactions, such as hyperkalemia induced by renin–angiotensin system inhibitors, opportunistic infections related to immunosuppression, metabolic disorders, and hepatotoxicity and nephrotoxicity [8,9,10,11,12,13]. Consequently, there is an urgent need to develop novel renoprotective agents that integrate multi-pathway synergistic regulation while ensuring safety.

Long-term clinical practice and experimental studies have confirmed that bioactive molecules derived from medicinal plants, as well as their synthetic or semi-synthetic derivatives, serve as essential lead compounds in the design of target-specific therapeutics. Notable examples include the natural anticancer agents paclitaxel (from *Taxus* species), vincristine and vinblastine (from *Catharanthus roseus*), and camptothecin (from *Camptotheca acuminata* Decne) together with its semi-synthetic derivative irinotecan; the marine-derived antineoplastic trabectedin (from *Ecteinascidia turbinata*); and the antimalarial artemisinin (from *Artemisia annua*) [14,15,16,17,18]. These precedents underscore that medicinal plants constitute a critical reservoir for the development of therapeutics targeting kidney disorders. Radix scutellariaem, the dried root of the perennial herb *Scutellaria baicalensis* Georgi, is commonly used in traditional Chinese medicine in clinical practice and was first systematically documented in the “Shennong Bencao Jing”, where it is noted for its effects in “clearing heat and dampness, purging fire, and detoxifying” [19,20]. Numerous pharmacological studies have gradually elucidated that the renoprotective effects of *S. baicalensis* are closely associated with its abundant natural flavonoids, particularly baicalein, baicalin, wogonin, and wogonoside. These natural flavonoids possess unique chemical structures characterized by ortho-phenolic hydroxyl groups and C-ring unsaturated ketones, which can contribute to antioxidant properties and facilitate electron transfer capabilities. Consequently, they play a crucial role in regulating redox balance, inflammatory responses, and cell apoptosis [21,22,23,24]. This provides a scientific foundation for developing therapeutic strategies targeting kidney diseases based on the active components derived from *S. baicalensis*.

For this review, we systematically searched Web of Science, PubMed, and CNKI. After full-text screening, studies were excluded if they (i) lacked topical relevance, (ii) were outdated or redundant, or (iii) had flawed designs or incomplete datasets. We critically assessed the renoprotective potential of *S. baicalensis*, focusing on the bioactive constituents baicalein, wogonin, and baicalin, and elucidated their pharmacological mechanisms in renal protection, including antioxidant effects, anti-inflammatory pathways, apoptosis regulation, and fibrosis mitigation. The synthesized evidence provides mechanistic insights into *S. baicalensis*-mediated interventions for kidney disease while highlighting their therapeutic prospects. This work establishes a foundation for advancing translational research on therapeutics derived from *S. baicalensis* and supports the development of standardized phytopharmaceuticals for renal pathologies.

## 2. Main Components of *S. baicalensis* for Renoprotective Effects

**Figure 1:**
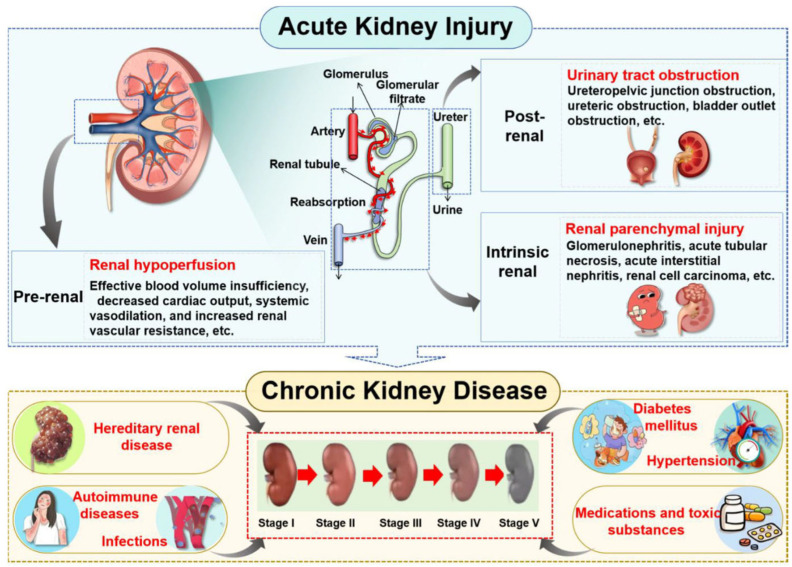
The forms and progression of kidney disease. As the primary organs responsible for urine production, the kidneys play a critical role in filtering blood to eliminate metabolic waste products and excess fluid, thus maintaining systemic fluid homeostasis and electrolyte balance. Renal injury can compromise kidney function and ultimately lead to the development of kidney disease. In clinical practice, kidney disease is broadly categorized into AKI and CKD. A variety of etiological factors can precipitate AKI, which, if unresolved or recurrent, may progress to CKD over time.

The kidney is an essential organ responsible for excreting waste in urine, playing a pivotal role in maintaining homeostasis and eliminating metabolic waste through the coordinated processes of glomerular filtration, tubular reabsorption, and endocrine secretion [25]. Kidney disease can be classified into two distinct forms, namely AKI and CKD, and each of them involves complex pathophysiological mechanisms (Figure 1). AKI is a clinical syndrome characterized by a rapid decline in renal function, which can be categorized into three types based on the different sites of etiological action: pre-renal, intrinsic renal, and post-renal [26,27,28]. Pre-renal AKI primarily results from renal hypoperfusion, commonly triggered by conditions such as hypovolemia (e.g., traumatic hemorrhage, surgical blood loss, severe diarrhea, or burns), reduced cardiac output (e.g., hemorrhagic shock), renal vasoconstriction, or mechanical obstruction of the renal artery [29,30,31]. Intrinsic renal AKI involves direct parenchymal damage frequently induced by nephrotoxic agents (e.g., contrast media or aminoglycosides), heavy metal exposure, or structural lesions affecting glomeruli or tubulointerstitial tissues [32,33]. Post-renal AKI typically arises from urinary tract obstruction with common causes including urolithiasis, neoplastic masses, and benign prostatic hyperplasia [34].

In contrast, the pathogenesis of CKD is predominantly associated with persistent metabolic dysregulation and systemic disorders. Major contributors to CKD include diabetic nephropathy, hypertensive nephropathy, autoimmune-mediated nephropathies (such as lupus nephritis), hyperuricemia (HUA), and drug-induced nephrotoxicity [35,36]. Furthermore, genetic anomalies such as polycystic kidney disease and Alport syndrome represent significant etiological factors in the development of CKD [37,38].

Epidemiological evidence illustrates a bidirectional interconversion between AKI and CKD. Prolonged AKI lasting beyond three months often progresses into CKD through maladaptive repair mechanisms. Conversely, patients with pre-existing CKD are susceptible to rapid deterioration of renal function due to acute triggers such as infection or hemorrhage, which can manifest as superimposed AKI—thereby accelerating progression to end-stage renal disease (ESRD) [39,40,41,42]. This reciprocal relationship establishes both conditions as critical determinants in the progression toward ESRD. Given the multidimensional pathological network characteristics inherent to kidney diseases, natural drugs exhibiting multi-target intervention features present unique advantages.

Renal diseases are orchestrated by a multidimensional pathological network that has traditionally been managed clinically with empirical combinations of renin–angiotensin–aldosterone system inhibitors, immunosuppressants, and corticosteroids to slow progression, while pre-clinical research has largely pursued “multi-target” interventions. These unspecific strategies often disrupt physiological homeostasis and exhibit poorly predictable dose–response relationships. Consequently, therapeutic development is transitioning toward precision paradigms that integrate (i) molecular-targeted modulation of critical signaling nodes, (ii) cell-type-restricted interventions, and (iii) spatiotemporal adjustment of targets according to disease stage. The emerging “component–target” specificity framework provides a new roadmap for complex disorders, coupling target deconvolution technologies, ligand optimization, targeted delivery systems, and individualized regimens to convert conventional multi-target approaches into precise, target-directed control. Medicinal plants, with their extensive chemodiversity, constitute an invaluable repository for identifying nephro-specific targets and are poised to accelerate the clinical translation of this precision strategy, offering patients more effective and safer therapeutic options.

*S. baicalensis*, a natural medicinal plant, contains various kinds of bioactive components, including flavonoids, volatile oils, polysaccharides, alkaloids, terpenoids, and trace elements [43]. Numerous studies have demonstrated that flavonoids serve as the primary constituents of *S. baicalensis* to exert multiple pharmacological effects including renoprotection. A total of 125 flavonoids have been isolated from *S. baicalensis*, comprising 68 free flavonoid aglycones and 57 flavonoid glycosides [44,45,46,47,48]. The flavonoids present in *S. baicalensis* are responsible for the renoprotective activities, especially baicalein, wogonin, baicalin, and wogonoside. The fundamental characteristics of the four bioactive molecules are presented in Table 1.

For these flavonoids, aglycones such as baicalein and wogonin exhibit favorable lipophilicity, whereas their corresponding glycosides baicalin and wogonoside exhibit a certain degree of water solubility. Given that baicalein, wogonin, baicalin, and wogonoside all contain phenolic hydroxyl groups, they display weak acidic properties. As thus, for the extraction of these flavonoids from *S. baicalensis*, ethanol solutions of appropriate concentration can be employed, or alternatively, the alkali dissolution–acid precipitation method may be utilized. Yan et al. employed ultrasonic-assisted extraction to prepare flavonoids from the stems and leaves of *S. baicalensis*, finding that the total flavonoid content could reach 24.43 mg·g^−1^ under the optimized conditions: 55% ethanol concentration, a solid-to-liquid ratio of 1:50, an extraction duration of 60 min, and a temperature of 50 °C [49]. Jiang et al. investigated the application of the alkali dissolution–acid precipitation technique and demonstrated that both baicalin and baicalein can be effectively extracted from *S. baicalensis*. Subsequent acid hydrolysis enabled further purification, yielding baicalein with a purity of 99.35% [50].

Notably, the low solubility of flavonoids from *S. baicalensis* may result in suboptimal bioavailability. To address this issue, a solvent-evaporation method was employed to prepare a baicalein solid dispersion, achieving a 35.1-fold enhancement in apparent solubility compared to the raw drug, with excellent physicochemical stability maintained over 80 days [51]. In parallel, baicalein-*β*-cyclodextrin-grafted-chitosan nanoparticles with a narrow size distribution (mean diameter ≈ 424.5 nm) were prepared [52]. MIC determination against *Staphylococcus aureus* showed a 50% reduction in the minimum inhibitory concentration (12.5 µg·mL^−1^ versus 25 µg·mL^−1^ for free baicalein), and colony-count assays corroborated the superior bactericidal efficacy of the nanoparticulate formulation. Nanotechnology-based platforms have enabled the development of a series of baicalin nanoformulations, including nanoparticles, nanoliposomes, and nanoemulsions, which not only enhance the aqueous solubility of baicalin but confer size-dependent targeting capabilities [53,54,55]. In one study, a novel crystalline solid lipid nanoparticle system loaded with baicalin was prepared via coacervation using stearic acid alkaline salt as the lipid matrix [56]. These solid lipid nanoparticles significantly improved the oral bioavailability of baicalin, with the area under the concentration–time curve and maximum plasma concentration being 2.58-fold and 1.61-fold higher, respectively, than those of the free drug. This provides a novel strategy for addressing the poor aqueous solubility and low oral bioavailability of flavonoids.

## 3. Effects and Mechanisms of *S. baicalensis* Against AKI

**Figure 2:**
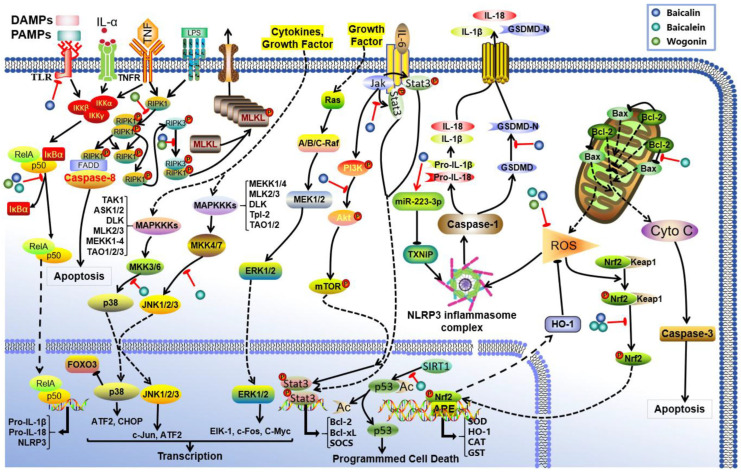
Mechanism of action of *S. baicalensis* against AKI. Baicalin, baicalein, and wogonin have been identified to inhibit AKI through multiple molecular pathways. Baicalin attenuates AKI by modulating oxidative stress via the miR-223-3p-TXNIP/NLRP3 pathway, suppressing TLR-induced inflammation, and activating the Nrf2/ARE, PI3K/Akt, and JAK2/STAT3 signaling cascades. Baicalein ameliorates AKI predominantly by inhibiting MAPK and NF-*κ*B signaling, enhancing the Nrf2/HO-1-mediated antioxidant response, reducing reactive oxygen species (ROS) production, and modulating apoptosis via the Bcl-2/Bax and SIRT1 pathways. Wogonin exerts protective effects against AKI via dual-level regulation of the NF-*κ*B and RIPK1 signaling axes.

AKI is a multifactorial clinical syndrome characterized by complex pathological mechanisms that are intricately associated with the activation of inflammatory cascades, imbalances in oxidative stress, and various forms of cell death, including necrosis, apoptosis, autophagic cell death, and ferroptosis [57]. These pathological alterations ultimately lead to renal damage and an irreversible decline in renal function [34,58]. Recent studies have demonstrated that baicalin, baicalein, and wogonin intervene in the AKI process through a multi-target mechanism (Figure 2).

Studies have elucidated that the renoprotective effects of baicalin are primarily manifested in the following pathways: (1) Antioxidant effects. Baicalin activates the Nrf2/HO-1 pathway and enhances the expression of miR-223-3p, which inhibits the TXNIP/NLRP3 pathway, resulting in reduced production of malondialdehyde (MDA) and myeloperoxidase while simultaneously increasing the activity of antioxidant enzymes, including superoxide dismutase (SOD) and glutathione [59,60,61,62,63]. (2) Anti-inflammatory effects. Baicalin exerts anti-inflammatory effects via several pathways, including TLR2/4, Nrf2/ARE, PI3K/Akt, and JAK2/STAT3, by effectively reducing levels of inflammatory factors such as IL-1*β* and TNF-*α* while inhibiting NF-*κ*B activation [64,65,66,67,68,69]. (3) Anti-apoptotic effects. Baicalin can regulate Klotho protein levels along with members of the caspase family (e.g., caspase-3 and caspase-11), thereby maintaining balance between Bcl-2/Bax proteins [64,70,71,72,73,74]. Additionally, baicalin intervenes in both the Ros/NLRP3/Caspase-1/Gsdmd and NLRP3/caspase-1 pathways [75], achieving dual inhibition of apoptosis and pyroptosis.

Researchers have also found that another flavonoid baicalein exhibits renoprotective effects in AKI through three mechanisms. Firstly, it alleviates inflammatory responses by inhibiting the production of pro-inflammatory mediators such as TNF-*α* and IL-1*β*; this process is regulated via the dual suppression of MAPK phosphorylation cascades and NF-*κ*B nuclear translocation, alongside modulation of the Nrf2/HO-1 pathway [76,77,78,79,80]. Secondly, it mitigates oxidative stress damage by enhancing endogenous antioxidant defense systems, effectively decreasing ROS and reducing the generation of oxidative biomarkers such as advanced oxidative protein products and MDA [76,80,81]. Thirdly, baicalein modulates apoptotic pathways through coordinated regulation of the Bcl-2/Bax protein balance and SIRT1-mediated deacetylation of p53 [78,82]. Interestingly, emerging evidence reveals the anti-ferroptotic activity of baicalein by modulating arachidonate 12-lipoxygenase [83]. In contrast to baicalein’s multimodal mechanisms, wogonin mainly targets programmed necrosis through dual-level regulation of the NF-*κ*B and RIPK1 signaling axis [84,85,86]. This mechanism involves concurrent transcriptional control and post-translational modifications to suppress inflammatory necrosis in renal cells. The differential targets exhibit preference between these two flavonoids. Baicalein mainly improves AKI via apoptosis/ferroptosis regulation, while wogonin treats AKI via necrotic pathways; this demonstrates their complementary therapeutic potential in managing AKI.

## 4. Effects and Mechanism of *S. baicalensis* Against CKD

### 4.1. Diabetic Kidney Disease (DKD)

DKD is a leading cause of ESRD, and its pathology is intricately linked to structural and functional damage to the renal microvasculature resulting from disorders in glucose metabolism [87]. For patients with DKD, clear pathological changes can be observed in their kidneys, including thickening of the glomerular basement membrane, proliferation of the extracellular matrix (ECM), and impairment of microvascular function [88,89]. Recent studies have demonstrated that *S. baicalensis* extracts and their bioactive components such as baicalin and wogonin can mitigate the progression of DKD through a multi-target regulatory mechanism (Table 2).

Baicalin has been demonstrated to possess significant anti-inflammatory and antioxidant effects through targeting multiple pathways. At the molecular level, baicalin activates the Nrf2 signaling pathway, specifically involving Keap1/Nrf2/ARE regulation, to promote the expression of antioxidant enzymes. Concurrently, baicalin inhibits pro-inflammatory signaling pathways such as MAPK and SphK1/S1P/NF-*κ*B to mitigate inflammatory responses [90,91]. Additionally, baicalin can suppress miR-141 expression to activate the Sirt1/Nrf2 signaling axis, thereby establishing a multi-pathway synergistic network [92]. As a result, the levels of pro-inflammatory cytokines, including IL-1*β*, IL-6, and TNF-*α*, as well as oxidative stress markers such as MDA and 8-hydroxydeoxyguanosine, can be significantly altered by baicalin. Additionally, baicalin can upregulate gene expression of CAT and Mn-SOD, along with promoting forkhead box O3a protein expression, which effectively ameliorates oxidative stress in damaged renal tissues [93]. Moreover, baicalin employs a dual inhibition strategy to intervene in the pathological processes associated with DKD. Baicalin can also decrease PKC activity alongside the expression of phosphorylated connexin 43, and reduce ECM production, TGF-β1 expression, and the production of advanced glycation end products and VEGF [94,95,96,97]. This multi-pathway coordination can effectively reverse DKD-related pathological damage via both metabolic modulation and vascular repair. Furthermore, baicalin can improve lipid metabolism disorders associated with DKD by specifically targeting FK506-binding protein 51 [98].

The molecular regulatory mechanisms of baicalein and wogonin in DKD has been further elucidated by some experimental studies. Baicalein was demonstrated to inhibit the activity of 12/15-lipoxygenase (12/15-LO) and AMPKα, thereby modulating glucose metabolism by suppressing anabolic processes while promoting catabolic pathways [99,100]. Additionally, it attenuates the hs-CRP/FC-*γ*R signaling axis and significantly reduces the overexpression of inducible iNOS and TGF-*β*1 mediated by NF-κB, thus alleviating renal inflammatory responses [99,101]. Wogonin can synergistically regulate various signaling pathways including TLR/NF-*κ*B, PI3K/Akt/NF-*κ*B, and KLF4/NF-*κ*B [102,103,104], which are critical for modulating inflammatory responses. It also inhibits the macrophage signaling cascade DKDM3OS/KLF4 while balancing Bcl-2-mediated apoptosis and autophagy [105,106,107], effectively safeguarding podocyte structure and function. Notably, wogonin’s inhibitory effects on the TLR4-JAK/STAT/AIM2 signaling axis and MRP8 expression present new therapeutic targets for mitigating pathological damage in DKD [106]. As another active component in *S. baicalensis*, wogonoside targets both the NF-*κ*B p65-MMP28 and HNF4A-NRF2 signaling axes, and this dual action exerts good inhibitory effects on inflammatory responses as well as oxidative stress during the pathological progression of DKD [106,108].

### 4.2. Hyperuricemia

HUA is a metabolic disorder primarily characterized by dysfunction in purine metabolism, leading to an imbalance between the overproduction of uric acid and its impaired excretion [109]. Notably, approximately 90% of clinical cases are attributed to compromised uric acid excretion function [110]. In this pathological state, supersaturated serum uric acid can crystallize into sodium urate crystals, which precipitate and deposit in the kidneys, thereby inducing severe renal inflammation [111,112].

Baicalin exhibits a multi-target anti-HUA mechanism. Firstly, baicalin regulates the Panx-1/P2X7 purinergic signaling pathway and the NLRP3 inflammasome-mediated classical pyroptosis pathway to effectively inhibit signal transduction in renal tubular epithelial cells activated by sodium urate crystals and thereby reduce the level of pyroptosis in kidney cells [113]. Secondly, baicalin competitively binds to the active site of xanthine oxidase to obstruct substrate binding of hypoxanthine/xanthine and significantly inhibit uric acid biosynthesis [114]. Thirdly, by downregulating the secretion of lactate dehydrogenase, nitric oxide, and pro-inflammatory factors such as TNF-*α* and IL-1*β*, baicalin synergistically modulates oxidative stress (resulting in reduced ROS levels) and apoptosis pathways, thus alleviating damage to renal tubular epithelial cells induced by sodium urate crystals [114]. Furthermore, baicalin can regulate the PI3K/Akt/NF-*κ*B signaling axis to achieve an integrated effect that combines xanthine oxidase inhibition with inflammation relief and apoptosis blockade through cross-regulation of the TLR/NLRP3/NF-*κ*B and MAPK pathways [115,116].

Baicalein was demonstrated to have a renoprotective effect through a dual mechanism. Firstly, it directly inhibits the activity of xanthine oxidoreductase, thereby reducing uric acid production. Secondly, it was found to enhance the excretion of uric acid in a hyperuricemic model mice [117,118]. This combined action ultimately alters serum uric acid levels and improves renal function.

### 4.3. Renal Fibrosis (RF)

RF represents the primary pathological feature of CKD as it progresses to its end stage. This condition is characterized by the loss of functional nephrons, abnormal proliferation of fibroblasts, and excessive deposition of ECM. These changes lead to glomerulosclerosis, tubular atrophy, and interstitial fibrosis, ultimately culminating in irreversible renal failure [119,120,121]. Recent pharmacological studies have demonstrated that the active components derived from *S. baicalensis* exhibit significant advantages in multi-target therapy for combating organ fibrosis. The methanol extract of *S. baicalensis* has been shown to exert anti-fibrotic effects by modulating TGF-β1-mediated activation of renal fibroblasts and intervening in oxidative stress pathways [122]. Furthermore, the total aglycone extract can downregulate the expression of ECM-related genes, thereby preventing the progression of renal interstitial fibrosis [123].

**Figure 3:**
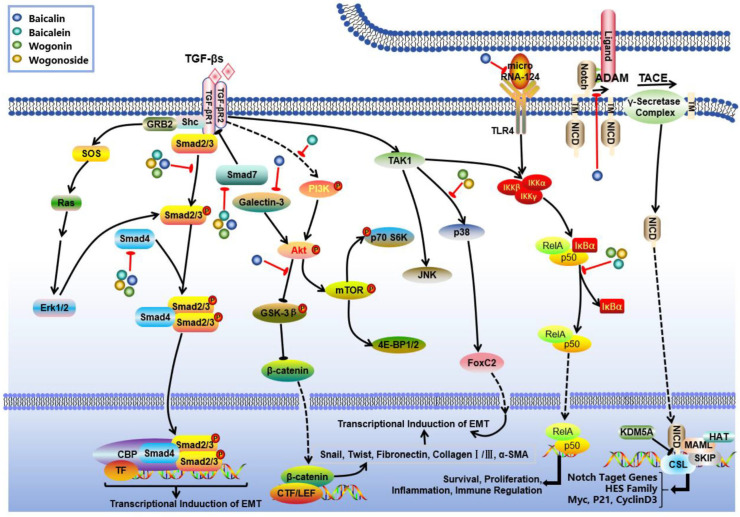
Mechanism of action of *S. baicalensis* against RF. Baicalein, baicalin, and wogonin were found to modulate the progression of RF through synergistic effects across multiple pathways. Baicalein can inhibit fibroblast proliferation and ECM deposition via TGF-β/Smad, PI3K/Akt, and MAPK. Baicalin suppresses RF by regulating TGF-*β*-mediated Smad and Notch1, microRNA-124/TLR4/NF-*κ*B and Galectin-3/Akt/GSK-3β/Snail signaling pathways. Wogonin and its glycoside derivatives exhibit a potent inhibitory effect on RF via the inhibition of the TGF-*β*-associated p38MAPK and Smad branches as well as the NF-*κ*B axes.

In-depth mechanistic studies have elucidated that flavonoids such as baicalein, baicalin, and wogonin can modulate the progression of RF through synergistic effects across multiple pathways (Figure 3). Specifically, baicalein exhibits a good inhibitory effect on RF. Baicalein was demonstrated to inhibit fibroblast proliferation and ECM deposition via several signaling axes, including TGF-*β*/Smad, PI3K/Akt, and MAPK [124,125,126]. Furthermore, it counteracts renal ferroptosis by upregulating the expression of ferroptosis-related proteins such as SLC7A11, GPX4, and FTH [127]. Additionally, baicalein can enhance the survival of renal tubular epithelial cells while mitigating interstitial fibrosis by regulating the balance of endoplasmic reticulum stress-related factors including caspase-3/9 and Bax/Bcl-2 [128].

According to some studies, another flavonoid, namely baicalin, also has obvious therapeutic effects on RF. Baicalin has been found to suppress RF at the molecular level by targeting multiple key nodes of the TGF-*β* signaling pathway. It downregulates Smad3 and upregulates Smad7 expression through modulation of the TGF-*β*/Smad signaling axis [129,130,131]. Additionally, baicalin indirectly regulates TGF-*β*1 levels via the Notch1 signaling pathway or by reducing plasma Angiotensin II content [132,133]. Moreover, baicalin inhibits renal tubular epithelial cell trans-differentiation and ECM deposition [124], thereby delaying the progression of renal tubular fibrosis through regulation of the microRNA-124/TLR4/NF-*κ*B signaling pathway and enhancement of CPT1*α*-mediated fatty acid oxidation [134,135,136]. For glomerular fibrosis, baicalin reduces *α*-smooth muscle actin (*α*-SMA) levels by inhibiting TGF-*β*1 expression while also decreasing advanced glycation end products and connective tissue growth factor expression. This action contributes to improvements in diabetic nephropathy-related glomerular lesions [95,96,137,138]. Furthermore, baicalin demonstrates multi-target characteristics in overall kidney protection by regulating the Galectin-3/Akt/GSK-3β/Snail signaling pathway [139]; reversing inhibitory effect of DNA hypermethylation on the Klotho gene [140]; and significantly alleviating glomerular, tubular, and interstitial fibrosis in CKD model rats. The mechanism for improving mitochondrial function involves enhancing CPT1*α*-mediated fatty acid oxidation [135]. Additionally, it was found to attenuate renal interstitial fibrosis by inhibiting NF-*κ*B nuclear translocation as well as STAT3 phosphorylation processes [141].

In addition, wogonin and its glycoside derivatives exhibit a potent inhibitory effect on RF through a multi-target synergistic mechanism. The primary mechanisms involve the modulation of inflammation-related signaling pathways and the regulation of pro-fibrogenic factors. Research has demonstrated that these compounds significantly inhibit the TGF-*β*1 signaling pathway, including both the p38MAPK and Smad3 branches as well as the NF-*κ*B signaling pathway and the TLR4/MAPK axis, thereby establishing a coordinated regulatory network across multiple inflammatory pathways [138,142,143,144]. At the molecular level, they effectively downregulate the expression of fibrogenic markers such as fibronectin, *α*-SMA, collagen I, E-cadherin, and TGF-*β*1 while concurrently modulating oxidative stress-related enzymes such as CAT and SOD to mitigate oxidative damage [145]. Furthermore, these components inhibit the release of pro-inflammatory cytokines including TNF-*α* and IL-1*β* and alleviate endoplasmic reticulum stress, thereby delaying apoptosis in renal tubular epithelial cells [145]. This multifaceted mechanism not only reduces inflammatory infiltration but inhibits abnormal ECM deposition. In all, these regulatory effects can contribute to ameliorating glomerulosclerosis and renal tubulointerstitial fibrosis and ultimately preserve renal parenchymal structure.

### 4.4. Renal Cell Carcinoma

Renal cell carcinoma (RCC), a malignant neoplasm originating from the renal tubular epithelium, accounts for approximately 90% of all renal malignancies and is recognized as one of the most aggressive tumor types within the urinary system. Contemporary pharmacological research has demonstrated that substances from *S. baicalensis* exhibit significant anti-tumor activity. Ethanol extract of *S. baicalensis* demonstrated tumor proliferation by inducing G2/M phase arrest in the human renal cancer cell line Caki-1. Network pharmacology analyses indicate that the core components in *S. baicalensis* exert anti-RCC effects through multiple signaling pathways, including PI3K-Akt, Ras, MAPK, p53, VEGF, and JAK-STAT [146]. Some investigations have elucidated that flavonoids repress RCC in various ways, specifically wogonin. Wogonin was demonstrated to inhibit tumor growth by interfering with cell cycle progression, inducing DNA damage stress responses, and activating apoptosis pathways [147]. Additionally, wogonin selectively induces programmed cell death in RCC cells by targeting the CDK4-RB signaling pathway [148]. These findings provide compelling experimental evidence supporting the potential application of *S. baicalensis* and its active components in the treatment of renal cancer.

## 5. Conclusions and Future Perspectives

*S. baicalensis*, a natural medicinal plant with abundant therapeutic properties, has a variety of bioactive compounds, such as flavonoids, terpenoids, and polysaccharides. Notably, flavonoids such as baicalein and wogonin, along with their glycosides, have been demonstrated to exert significant renoprotective effects. For AKI, *S. baicalensis* exerts robust renoprotective actions through multi-target synergistic mechanisms that include the inhibition of MAPKs/NF-*κ*B inflammatory signaling pathways, activation of the antioxidant defense system, regulation of the Bcl-2/Bax-SIRT1/p53 apoptosis pathway, and suppression of ferroptosis mediated by 12-lipoxygenase. Among these mechanisms, the modulation of ferroptosis represents a novel therapeutic avenue for AKI treatment. In CKD, the active components derived from *S. baicalensis* can enhance renal function by delaying the progression of fibrosis through the inhibition of the TGF-*β*/Smad signaling pathway and cell injury induced by oxidative stress. Nevertheless, current evidence primarily originates from animal studies; therefore, further clinical validation is essential to confirm their translational potential for therapeutic applications.

Despite these promising findings, several significant challenges persist within the current research landscape.

First, current studies predominantly concentrate on flavonoids as the bioactive ingredients from *S. baicalensis* for the treatment of kidney disease, with insufficient exploration of other structurally diverse components such as terpenoids and polysaccharides. Second, there is a substantial body of research focusing on the inhibitory effects on renal tubular injury, but there is a lack of investigations concerning glomerular injury. Thirdly, the intricate regulatory network underlying the synergistic nephroprotective effects of multiple components necessitate further elucidation through systems biology approaches. Fourth, the strategy for clinical evaluation of the active components of *S. baicalensis* has yet to be established.

To address these challenges, future research is necessary to systematically explore the renoprotective effects and underlying mechanisms. Firstly, the renoprotective effects of other types of compounds in *S. baicalensis,* such as terpenoids and polysaccharides, should be further explored. Secondly, the integration of multi-omics technologies, including spatial transcriptomics and protein–protein interaction network analysis, can be employed to facilitate a comprehensive mechanistic understanding of the “component–target–pathway” regulatory network. Thirdly, advanced models such as organoids and organ-on-a-chip platforms should be used to enhance the translational value in new drug development. Furthermore, special attention should be directed towards creating novel nano-drug delivery systems to increase the therapeutic effects of molecules from *S. baicalensis* to overcome its bioavailability limitations through formulation innovations. These systematic and multidisciplinary approaches will effectively promote the application of *S. baicalensis* in treating kidney disease.

## Figures and Tables

**Table 1:** Main bioactive flavonoids in *S. baicalensis* that exert renoprotective effects.

Compound	Molecular Structure	CAS	Molecular Formula	Molecular Weight	Plant Part
Baicalein	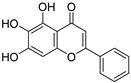	491-67-8	C_15_H_10_O_5_	270.237	Root; Hairy Root
Wogonin	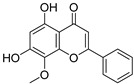	632-85-9	C_16_H_12_O_5_	284.263	Root; Aerial Part Hairy Root
Baicalin	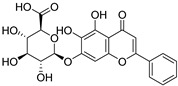	21967-41-9	C_21_H_18_O_11_	446.361	Root; Aerial Part Hairy Root
Wogonoside	** 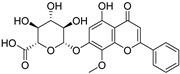 **	51059-44-0	C_22_H_20_O_11_	460.388	Root; Hairy Root

**Table 2:** Bioactive components of *S. baicalensis* and their mechanisms of action against DKD.

Compound	In Vivo or In Vitro Models	Mechanism of Action	Outcome	Ref.
Baicalin	db/db and db/m mouse model; palmitic acid-induced MPC-5 cells; high-glucose-induced HK-2 cells	Activation of Nrf2 signaling pathway; inhibition of MAPK and SphK1/S1P/NF-*κ*B signaling pathway, as well as inhibition of the expression of miR-141 to activate the Sirt1/Nrf2 signaling axis	Oxidative stress ↓ Inflammation ↓ Apoptosis ↓	[90,91]
	STZ-induced DKD mouse model; high-glucose-induced SV40-Mes-13 cells	Down-regulation of the expression of miR-141 to activate the Sirt1/Nrf2 signaling axis	Oxidative stress ↓ Apoptosis ↓	[92]
	STZ combined with high fat and high-glucose-induced DKD mouse model	Down-regulation the expression of MDA and 8-hydroxydeoxyguanosine; up-regulation of the expression of the genes encoding CAT and Mn-SOD, as well as forkhead box O3a protein	Oxidative stress ↓	[93]
	STZ-induced and STZ combined with high-fat-induced DKD rat model	Inhibition of the activity of PKC and the expression of phosphorylated connexin 43 to decrease the content of ECM and TGF-*β*1; inhibition of the production of advanced glycation end products and the expression of VEGF	Inflammation ↓ ECM production ↓	[94,95,96,97]
	High-fat-diet/STZ-induced DKD mouse model	Targeting FK506-binding protein 51	Lipid metabolism ↑	[98]
Baicalein	STZ combined with high-fat and high-glucose-induced DKD rat model	Down-regulation the expression of AMPK*α*, hs-CRP and Fc*γ*R	Inflammation ↓	[99]
	STZ-induced DKD model in C57BL/6J (WT) and 12/15-LO-deficient mice	Down-regulation the activity of 12/15-LO	Inflammation ↓	[100]
	STZ combined with high-fat -induced DKD rat model	Inhibition of NF-*κ*B signaling pathway to down-regulation the expression of iNOS and TGF-*β*1	Lipid and glucose metabolism ↑ Oxidative stress ↓ Inflammation ↓	[101]
Wogonin	STZ combined with high-fat-induced DKD rat model	Inhibition of TLR/NF-*κ*B signaling pathway	Inflammation ↓	[102]
	STZ-induced DKD mouse model; high-glucose-induced HK-2 cells	Inhibition of PI3K/Akt/NF-*κ*B signaling pathway	Autophagy ↓ Inflammation ↓	[103]
	High-glucose-induced RAW264.7 cells	Regulation of KLF4/NF-*κ*B signaling pathway in macrophages	Inflammation ↓	[104]
	db/db and db/m mouse model; high-glucose-induced RAW264.7 cells	Inhibition DNM3OS/KLF4 signaling pathway in macrophages	Oxidative stress ↓ Inflammation ↓	[105]
	STZ-induced DKD mouse model; high-glucose-induced podocyte cells	Regulation of the balance between apoptosis and autophagy mediated by Bcl-2	Apoptosis ↓ Autophagy ↓	[106,107]
	db/db mouse model; high-glucose-induced HK-2 cells	Inhibition of TLR4-mediated JAK/STAT/AIM2 signaling pathway	Apoptosis ↓ Autophagy ↓	[106]
Wogonoside	HFD/STZ-induced DKD mouse model; high-glucose-induced MPC-5 cells	Targeting the NF-*κ*B p65-MMP28 axis	Apoptosis ↓ Autophagy ↓	[106]
	STZ combined with high-fat-induced DKD mouse model; high-glucose-induced TCMK-1 cells	Targeting the HNF4A-NRF2 axis	Oxidative stress ↓	[108]

CAT: catalase; VEGF: vascular endothelial growth factor; ↓: downregulation; ↑: upregulation.

## Data Availability

The original contributions presented in this study are included in the article. Further inquiries can be directed to the corresponding author.

## Abbreviations

The following abbreviations are used in this manuscript:

AKI	Acute kidney injury
ALOX12	Arachidonate 12-lipoxygenase
CAT	Catalase
CKD	Chronic kidney disease
DKD	Diabetic kidney disease
DM	Diabetes mellitus
ECM	Extracellular matrix
ESRD	End-stage renal disease
HUA	Hyperuricemia
12/15-LO	12/15-Lipoxygenase
MDA	Malondialdehyde
RCC	Renal cell carcinoma
RF	Renal fibrosis
*S. baicalensis*	*Scutellaria baicalensis*
SOD	Superoxide dismutase
VEGF	Vascular endothelial growth factor
*α*-SMA	*α*-Smooth muscle actin

## References

[1] Menon S., Symons J.M., Selewski D.T. (2023). Acute kidney injury. Pediatr. Rev..

[2] Jager K.J., Kovesdy C., Langham R., Rosenberg M., Jha V., Zoccali C. (2019). A single number for advocacy and communication-worldwide more than 850 million individuals have kidney diseases. Nephrol. Dial. Transplant..

[3] Hall R.K., Kazancıoğlu R., Thanachayanont T., Wong G., Sabanayagam D., Battistella M., Ahmed S.B., Inker L.A., Barreto E.F., Fu E.L. (2024). Drug stewardship in chronic kidney disease to achieve effective and safe medication use. Nat. Rev. Nephrol..

[4] Hu H., Li W., Hao Y., Peng Z., Zou Z., Wei J., Zhou Y., Liang W., Cao Y. (2024). The SGLT2 inhibitor dapagliflozin ameliorates renal fibrosis in hyperuricemic nephropathy. Cell Rep. Med..

[5] Pradhan N., Dobre M. (2023). Emerging preventive strategies in chronic kidney disease: Recent evidence and gaps in knowledge. Curr. Atheroscler. Rep..

[6] Piccoli G.B., Cederholm T., Avesani C.M., Bakker S.J.L., Bellizzi V., Cuerda C., Cupisti A., Sabatino A., Schneider S., Torreggiani M. (2023). Nutritional status and the risk of malnutrition in older adults with chronic kidney disease-implications for low protein intake and nutritional care: A critical review endorsed by ERN-ERA and ESPEN. Clin. Nutr..

[7] Ostermann M., Lumlertgul N., Jeong R., See E., Joannidis M., James M. (2025). Acute kidney injury. Lancet.

[8] Baker M., Perazella M.A. (2020). NSAIDs in CKD: Are they safe?. Am. J. Kidney Dis..

[9] Alicic R.Z., Neumiller J.J., Tuttle K.R. (2025). Combination therapy: An upcoming paradigm to improve kidney and cardiovascular outcomes in chronic kidney disease. Nephrol. Dial. Transplant..

[10] Ahmadi F., Hwang Y.J., Muanda F.T. (2024). Pros and cons of the use of fluoroquinolone antibiotics in patients with kidney disease. Nephrol. Dial. Transplant..

[11] Chade A.R., Bidwell G.L. (2022). Novel drug delivery technologies and targets for renal disease. Hypertension.

[12] Bhandari S., Ives N., Brettell E.A., Valente M., Cockwell P., Topham P.S., Cleland J.G., Khwaja A., El Nahas M. (2016). Multicentre randomized controlled trial of angiotensin-converting enzyme inhibitor/angiotensin receptor blocker withdrawal in advanced renal disease: The STOP-ACEi trial. Nephrol. Dial. Transpl..

[13] Perazella M.A. (2018). Pharmacology behind common drug nephrotoxicities. Clin. J. Am. Soc. Nephrol..

[14] Jordan M.A., Wilson L. (2004). Microtubules as a target for anticancer drugs. Nat. Rev. Cancer.

[15] Ishikawa H., Colby D.A., Seto S., Va P., Tam A., Kakei H., Rayl T.J., Hwang I., Boger D.L. (2009). Total synthesis of vinblastine, vincristine, related natural products, and key structural analogues. J. Am. Chem. Soc..

[16] Olatunde O.Z., Yong J., Lu C., Ming Y. (2023). A review on shikonin and its derivatives as potent anticancer agents targeted against topoisomerases. Curr. Med. Chem..

[17] Tu Y. (2011). The discovery of artemisinin (qinghaosu) and gifts from Chinese medicine. Nat. Med..

[18] Chuk M.K., Balis F.M., Fox E. (2009). Trabectedin. Oncologist.

[19] Zhao Q., Chen X.Y., Martin C. (2016). *Scutellaria baicalensis*, the golden herb from the garden of chinese medicinal plants. Sci. Bull..

[20] Hou F., Yu Z., Cheng Y., Liu Y., Liang S., Zhang F. (2022). Deciphering the pharmacological mechanisms of *Scutellaria baicalensis* Georgi on oral leukoplakia by combining network pharmacology, molecular docking and experimental evaluations. Phytomedicine.

[21] Pietta P.G. (2000). Flavonoids as antioxidants. J. Nat. Prod..

[22] Havsteen B.H. (2002). The biochemistry and medical significance of the flavonoids. Pharmacol. Ther..

[23] Heim K.E., Tagliaferro A.R., Bobilya D.J. (2002). Flavonoid antioxidants: Chemistry, metabolism and structure-activity relationships. J. Nutr. Biochem..

[24] Kim H.P., Son K.H., Chang H.W., Kang S.S. (2004). Anti-inflammatory plant flavonoids and cellular action mechanisms. J. Pharmacol. Sci..

[25] Takasato M., Er P.X., Chiu H.S., Maier B., Baillie G.J., Ferguson C., Parton R.G., Wolvetang E.J., Roost M.S., Chuva de Sousa Lopes S.M. (2015). Kidney organoids from human iPS cells contain multiple lineages and model human nephrogenesis. Nature.

[26] Khwaja A. (2012). KDIGO clinical practice guidelines for acute kidney injury. Nephron. Clin. Pract..

[27] Jacob J., Dannenhoffer J., Rutter A. (2020). Acute kidney injury. Prim. Care.

[28] Thadhani R., Pascual M., Bonventre J.V. (1996). Acute renal failure. N. Engl. J. Med..

[29] Adiyeke E., Ren Y., Guan Z., Ruppert M.M., Rashidi P., Bihorac A., Ozrazgat-Baslanti T. (2023). Clinical courses of acute kidney injury in hospitalized patients: A multistate analysis. Sci. Rep..

[30] Molitoris B.A. (2022). Low-flow acute kidney injury: The pathophysiology of prerenal azotemia, abdominal compartment syndrome, and obstructive uropathy. Clin. J. Am. Soc. Nephrol..

[31] Hoste E.A., Bagshaw S.M., Bellomo R., Cely C.M., Colman R., Cruz D.N., Edipidis K., Forni L.G., Gomersall C.D., Govil D. (2015). Epidemiology of acute kidney injury in critically ill patients: The multinational AKI-EPI study. Intensive Care Med..

[32] Perazella M.A. (2019). Drug-induced acute kidney injury: Diverse mechanisms of tubular injury. Curr. Opin. Crit. Care.

[33] Perazella M.A., Rosner M.H. (2022). Drug-induced acute kidney injury. Clin. J. Am. Soc. Nephrol..

[34] Kellum J.A., Romagnani P., Ashuntantang G., Ronco C., Zarbock A., Anders H.J. (2021). Acute kidney injury. Nat. Rev. Dis. Primers.

[35] Webster A.C., Nagler E.V., Morton R.L., Masson P. (2017). Chronic kidney disease. Lancet.

[36] Law J.P., Pickup L., Pavlovic D., Townend J.N., Ferro C.J. (2023). Hypertension and cardiomyopathy associated with chronic kidney disease: Epidemiology, pathogenesis and treatment considerations. J. Hum. Hypertens..

[37] Andrassy K.M. (2013). Comments on ‘kdigo 2012 clinical practice guideline for the evaluation and management of chronic kidney disease’. Kidney Int..

[38] Stevens P.E., Levin A. (2013). Evaluation and management of chronic kidney disease: Synopsis of the kidney disease: Improving global outcomes 2012 clinical practice guideline. Ann. Intern. Med..

[39] Levey A.S., Eckardt K.U., Dorman N.M., Christiansen S.L., Hoorn E.J., Ingelfinger J.R., Inker L.A., Levin A., Mehrotra R., Palevsky P.M. (2020). Nomenclature for kidney function and disease: Report of a kidney disease: Improving global outcomes (KDIGO) consensus conference. Kidney Int..

[40] Chawla L.S., Bellomo R., Bihorac A., Goldstein S.L., Siew E.D., Bagshaw S.M., Bittleman D., Cruz D., Endre Z., Fitzgerald R.L. (2017). Acute kidney disease and renal recovery: Consensus report of the acute disease quality initiative (ADQI) 16 workgroup. Nat. Rev. Nephrol..

[41] Neyra J.A., Chawla L.S. (2021). Acute kidney disease to chronic kidney disease. Crit. Care Clin..

[42] Chawla L.S., Eggers P.W., Star R.A., Kimmel P.L. (2014). Acute kidney injury and chronic kidney disease as interconnected syndromes. N. Engl. J. Med..

[43] Wang Z.L., Wang S., Kuang Y., Hu Z.M., Qiao X., Ye M. (2018). A comprehensive review on phytochemistry, pharmacology, and flavonoid biosynthesis of *Scutellaria baicalensis*. Pharm. Biol..

[44] Zhou X.Q., Liang H., Lu X.H., Cai S.Q., Wang B., Zhao Y.Y. (2009). Flavonoids from *Scutellaria baicalensis* and their bioactivities. J. Peking Univ. Health Sci..

[45] Zhao T., Tang H., Xie L., Zheng Y., Ma Z., Sun Q., Li X. (2019). *Scutellaria baicalensis* Georgi. (Lamiaceae): A review of its traditional uses, botany, phytochemistry, pharmacology and toxicology. J. Pharm. Pharmacol..

[46] Song J.W., Long J.Y., Xie L., Zhang L.L., Xie Q.X., Chen H.J., Deng M., Li X.F. (2020). Applications, phytochemistry, pharmacological effects, pharmacokinetics, toxicity of *Scutellaria baicalensis* Georgi. and its probably potential therapeutic effects on COVID-19: A review. Chin. Med..

[47] Wen H.Z., Xiao S.Y., Wang Y.M., Luo G.A. (2004). General situation of chemical constitutions anddrug-processing of *Scutellaria baicalensis* Georgi. Nat. Prod. Res. Dev..

[48] Ma W., Liu T., Ogaji O.D., Li J., Du K., Chang Y. (2024). Recent advances in Scutellariae radix: A comprehensive review on ethnobotanical uses, processing, phytochemistry, pharmacological effects, quality control and influence factors of biosynthesis. Heliyon.

[49] Yan J., Zhang S.F., Mao X.X., Xu C.X., Liu B. (2024). Optimization of extraction process of flavonoids from *Scutellaria baicalensis* stem and leaf by response surface methodology and its content determination. Hubei Agric. Sci..

[50] Jiang J.J., Dong H.R. (2008). Preparation and characterization of high-purity baicalein. J. Beijing Univ. Chem. Technol..

[51] Yu H., Chang J.S., Kim S.Y., Kim Y.G., Choi H.K. (2017). Enhancement of solubility and dissolution rate of baicalein, wogonin and oroxylin A extracted from Radix scutellariae. Int. J. Pharm..

[52] Zhang Z., Chen J., Zou L., Tang J., Zheng J., Luo M., Wang G., Liang D., Li Y., Chen B. (2022). Preparation, characterization, and staphylococcus aureus biofilm elimination effect of baicalein-loaded β-cyclodextrin-grafted chitosan nanoparticles. Int. J. Nanomed..

[53] Liu Y., Ma Y., Xu J., Chen Y., Xie J., Yue P., Zheng Q., Yang M. (2017). Apolipoproteins adsorption and brain-targeting evaluation of baicalin nanocrystals modified by combination of Tween 80 and TPGS. Colloids Surf. B Biointerfaces.

[54] Wei Y., Liang J., Zheng X., Pi C., Liu H., Yang H., Zou Y., Ye Y., Zhao L. (2017). Lung-targeting drug delivery system of baicalin-loaded nanoliposomes: Development, biodistribution in rabbits, and pharmacodynamics in nude mice bearing orthotopic human lung cancer. Int. J. Nanomed..

[55] Wu L., Bi Y., Wu H. (2018). Formulation optimization and the absorption mechanisms of nanoemulsion in improving baicalin oral exposure. Drug Dev. Ind. Pharm..

[56] Hao J., Wang F., Wang X., Zhang D., Bi Y., Gao Y., Zhao X., Zhang Q. (2012). Development and optimization of baicalin-loaded solid lipid nanoparticles prepared by coacervation method using central composite design. Eur. J. Pharm. Sci..

[57] Basile D.P., Anderson M.D., Sutton T.A. (2012). Pathophysiology of acute kidney injury. Compr. Physiol..

[58] Hoste E.A.J., Kellum J.A., Selby N.M., Zarbock A., Palevsky P.M., Bagshaw S.M., Goldstein S.L., Cerdá J., Chawla L.S. (2018). Global epidemiology and outcomes of acute kidney injury. Nat. Rev. Nephrol..

[59] Sun Y., Liu M.W., Zhao Y.H., Lu Y.X., Wang Y.A., Tong C.W. (2020). Baicalin attenuates lipopolysaccharide-induced renal tubular epithelial cell injury by inhibiting the TXNIP/NLRP3 signalling pathway via increasing miR-223-3p expression. J. Biol. Regul. Homeost. Agents.

[60] Shi J., Wu G., Zou X., Jiang K. (2019). Enteral baicalin, a flavone glycoside, reduces indicators of cardiac surgery-associated acute kidney injury in rats. Cardiorenal Med..

[61] Li Q.H., Zhang Y.L., Xie X.J., Guo H.X., Wang X.Y. (2021). Propofol’s protective effect enhancement by baicalin mediated activation of Nrf2/HO-1 pathway on renal ischemia-reperfusion injury rats. Acta Chin. Med..

[62] Lin M., Li L., Zhang Y., Zheng L., Xu M., Rong R., Zhu T. (2014). Baicalin ameliorates H2O2 induced cytotoxicity in HK-2 cells through the inhibition of ER stress and the activation of Nrf2 signaling. Int. J. Mol. Sci..

[63] Wang Y., Li X., Yan C., Xie L., Yang Y. (2023). Baicalin exhibits a protective effect against cisplatin-induced cytotoxic damage in canine renal tubular epithelial cells. Metabolites.

[64] Xu J., Li S., Jiang L., Gao X., Liu W., Zhu X., Huang W., Zhao H., Wei Z., Wang K. (2021). Baicalin protects against zearalenone-induced chicks liver and kidney injury by inhibiting expression of oxidative stress, inflammatory cytokines and caspase signaling pathway. Int. Immunopharmacol..

[65] Lin M., Li L., Li L., Pokhrel G., Qi G., Rong R., Zhu T. (2014). The protective effect of baicalin against renal ischemia-reperfusion injury through inhibition of inflammation and apoptosis. BMC Complement. Altern. Med..

[66] He Q., Sun X., Zhang M., Chu L., Zhao Y., Wu Y., Zhang J., Han X., Guan S., Ding C. (2022). Protective effect of baicalin against arsenic trioxide-induced acute hepatic injury in mice through JAK2/STAT3 signaling pathway. Int. J. Immunopathol. Pharmacol..

[67] Xing Z.G., Wang Y.Z., Jia D.Z., Li Q.J. (2022). Study on the mechanism of protective effect of baicalin on acute kidney injury in rats with crush syndrome. Mod. J. Integr. Tradit. Chin. West. Med..

[68] Ning X., Luo D., Chen Y., Shao Y., Xu J. (2023). Baicalin reduces renal inflammation in mesangial proliferative glomerulonephritis through activation of Nrf2/ARE and PI3K/AKT pathways. Discov. Med..

[69] Xi B.S., Huang P.Z., Tong Z.Y., Yao C.L., Hou Y.Y., Ji Y. (2011). The role of NF-κB in the protective effects of baicalin for septic rats’ kidneys. Chin. J. Clin. Med..

[70] Li Z.P., Gu J., Zhang Y.C., Yang Q.T., Liu M. (2023). Protective effect and mechanism of baicalin on acute kidney injury in duced by rhabdomyolysis syndrome in rats. Lab. Med. Clin..

[71] Jia Y.C., Yang C., Zheng L., Li J.W., Zeng Y.G., Xu M., Zhu T.Y., Rong R.M. (2019). Protective effect of baicalin on acute kidney injury induced by aristolochic acid in mice. Chin. J. Clin. Med..

[72] Zhu Y., Fu Y., Lin H. (2016). Baicalin inhibits renal cell apoptosis and protects against acute kidney injury in pediatric sepsis. Med. Sci. Monit..

[73] Le J., Fan H., Sun M., Zhu J. (2021). Protective effect and mechanism of baicalin on acute kidney injury in septic mice. Chin. Crit. Care Med..

[74] Wang Y., Jia Y., Yang X., Liang B., Gao H., Yang T. (2018). A potential role of baicalin to inhibit apoptosis and protect against acute liver and kidney injury in rat preeclampsia model. Biomed. Pharmacother..

[75] Li Y., Wang J., Huang D., Yu C. (2022). Baicalin alleviates contrast-induced acute kidney injury through ROS/NLRP3/Caspase-1/GSDMD pathway-mediated proptosis in vitro. Drug Des. Dev. Ther..

[76] Sahu B.D., Mahesh Kumar J., Sistla R. (2015). Baicalein, a bioflavonoid, prevents cisplatin-induced acute kidney injury by up-regulating antioxidant defenses and down-regulating the MAPKs and NF-κB pathways. PLoS ONE.

[77] Chen Y., Zheng Y., Zhou Z., Wang J. (2018). Baicalein alleviates tubular-interstitial nephritis in vivo and in vitro by down-regulating NF-κB and MAPK pathways. Braz. J. Med. Biol. Res..

[78] Lai C.C., Huang P.H., Yang A.H., Chiang S.C., Tang C.Y., Tseng K.W., Huang C.H. (2016). Baicalein, a component of *Scutellaria baicalensis*, attenuates kidney injury induced by myocardial ischemia and reperfusion. Planta Med..

[79] Hu Y., Lyu C.Y., Dai X., Wang Y.H., Zhao R.Z., Feng J.X., Lou S.l., Yan H., Sun C. (2025). Baicalein intervenes in the Nrf-2/HO-1 signaling pathway reduces kidney injury in sepsis mice. Chin. J. Vet. Sci..

[80] Dai C., Tang S., Wang Y., Velkov T., Xiao X. (2017). Baicalein acts as a nephroprotectant that ameliorates colistin-induced nephrotoxicity by activating the antioxidant defence mechanism of the kidneys and down-regulating the inflammatory response. J. Antimicrob. Chemother..

[81] Wu K., Li H., Tian J., Lei W. (2015). Protective effect of baicalein on renal ischemia/reperfusion injury in the rat. Ren. Fail..

[82] Yu M., Li H., Wang B., Wu Z., Wu S., Jiang G., Wang H., Huang Y. (2023). Baicalein ameliorates polymyxin B-induced acute renal injury by inhibiting ferroptosis via regulation of SIRT1/p53 acetylation. Chem. Biol. Interact..

[83] Guo S., Zhou L., Liu X., Gao L., Li Y., Wu Y. (2024). Baicalein alleviates cisplatin-induced acute kidney injury by inhibiting ALOX12-dependent ferroptosis. Phytomedicine.

[84] Meng X.M., Li H.D., Wu W.F., Ming-Kuen Tang P., Ren G.L., Gao L., Li X.F., Yang Y., Xu T., Ma T.T. (2018). Wogonin protects against cisplatin-induced acute kidney injury by targeting RIPK1-mediated necroptosis. Lab. Invest.

[85] Wang J.N., Wang J.J., Wang M.X., Wu Y.G., Qi X.M. (2024). Protective effect of wogonin on lipopolysaccharide-induced acute kidney injury in mice. Acta Univ. Med. Anhui.

[86] Qi Q., Li Y., Ding M., Huang C., Omar S.M., Shi Y., Liu P., Cai G., Zheng Z., Guo X. (2024). Wogonin inhibits apoptosis and necroptosis induced by nephropathogenic infectious bronchitis virus in chicken renal tubular epithelial cells. Int. J. Mol. Sci..

[87] Alicic R.Z., Rooney M.T., Tuttle K.R. (2017). Diabetic kidney disease: Challenges, progress, and possibilities. Clin. J. Am. Soc. Nephrol..

[88] Gupta S., Dominguez M., Golestaneh L. (2023). Diabetic kidney disease: An update. Med. Clin. N. Am..

[89] Thomas M.C., Brownlee M., Susztak K., Sharma K., Jandeleit-Dahm K.A., Zoungas S., Rossing P., Groop P.H., Cooper M.E. (2015). Diabetic kidney disease. Nat. Rev. Dis. Primers.

[90] Ma L., Wu F., Shao Q., Chen G., Xu L., Lu F. (2021). Baicalin alleviates oxidative stress and inflammation in diabetic nephropathy via Nrf2 and MAPK signaling pathway. Drug Des. Dev. Ther..

[91] Ren G., Jiao P., Yan Y., Ma X., Qin G. (2023). Baicalin exerts a protective effect in diabetic nephropathy by repressing inflammation and oxidative stress through the SphK1/S1P/NF-κB signaling pathway. Diabetes Metab. Syndr. Obes..

[92] Yin Q.Q., Xia Y.Y., Chen J., Xiao L., Wang Y., Zhao Y.Y., Ye G., Wu J. (2019). Baicalin improves diabetic nephropathy in mice by activating Sirt1/Nrf2 signal through miR-141 inhibition. Med. J. Wuhan Univ..

[93] Xi Y.L., Wang M., Zhou J. (2024). Baicalin regulate oxidative stress to prevent and treat diabetic nephropathy. Chin. J. Bioprocess. Eng..

[94] Zhao B.L., Li X.T., Pan S.M., Zhang X.N., Huang Z.J., Wu S.F., Zhang S.X., Lin R.S., Su N. (2016). Effect of baicalin capsules on kidney pathological morphology and protein kinase C in the rats of diabetic nephropath. World J. Integr. Tradit. West. Med..

[95] Zhang Y., Song G.H. (2003). The research of kidney-protective effects of erigeron breviscapus on diabetic nephropathy in rats. Chin. J. Diabetes.

[96] Hu S.H., Zhang M.X. (2007). The protective effect of baicalin on the kidney of STZ induced diabeti crats and its relation to VEGF. Chin. J. Hosp. Pharm..

[97] Zhao B.L. (2015). The effects of baicalin on phosphorylation sites on the Connexin43 gap junction protein in diabetic nephropathy rats kidney. Master’s Thesis.

[98] Li M., Zhu H.Y., Zhao S.Y., Li X.D., Tong S.M., Ma J., Xu A.J., Zhang J. (2025). Baicalin alleviates lipid metabolism disorders in diabetic kidney disease via targeting FKBP51. Phytomedicine.

[99] Sun P., Lu L., Chen J., Liu X.D., Zhang Q., Wang X. (2018). AMPKα, hs-CRP and FcγR in diabetic nephropathy and drug intervention. Exp. Ther. Med..

[100] Faulkner J., Pye C., Al-Shabrawey M., Elmarakby A.A. (2015). Inhibition of 12/15-lipoxygenase reduces renal inflammation and injury in streptozotocin-induced diabetic mice. J. Diabetes Metab..

[101] Ahad A., Mujeeb M., Ahsan H., Siddiqui W.A. (2014). Prophylactic effect of baicalein against renal dysfunction in type 2 diabetic rats. Biochimie.

[102] Hou Y.L., Mei W., Guo F.F., He M.J., Wu G.Q., Lin M. (2021). Effects of wogonin on blood glucose and renal tissue TLR4 and NF-κB p65 levels in rats with diabetic nephropathy. Guid. J. Tradit. Chin. Med. Pharm..

[103] Lei L., Zhao J., Liu X.Q., Chen J., Qi X.M., Xia L.L., Wu Y.G. (2021). Wogonin alleviates kidney tubular epithelial injury in diabetic nephropathy by inhibiting PI3K/Akt/NF-κB signaling pathways. Drug Des. Dev. Ther..

[104] Xie S., Xu X.X. (2022). Effect of wogonoside on KLF4/NF-κB signal pathway in RAW264.7 macrophages stimulated by high glucose. Act. Univ. Med. Anhui.

[105] Xie S. (2022). Wogonoside interferes with DNM3OS/KLF4 pathway and regulates macrophage activation in diabetic nephropathy. Master’s Thesis.

[106] Liu X.Q., Jiang L., Li Y.Y., Huang Y.B., Hu X.R., Zhu W., Wang X., Wu Y.G., Meng X.M., Qi X.M. (2022). Wogonin protects glomerular podocytes by targeting Bcl-2-mediated autophagy and apoptosis in diabetic kidney disease. Acta Pharmacol. Sin..

[107] Wang Y. (2020). Protective effect and mechanism of wogonin on diabetic kidney podocyte injury. Ph.D. Thesis.

[108] Li X., Zhao S., Li M., Xing X., Xie J., Wang M., Xu A., Zhao Q., Zhang J. (2025). Wogonoside ameliorates oxidative damage in tubular epithelial cells of diabetic nephropathy by modulating the HNF4A-NRF2 axis. Int. Immunopharmacol..

[109] Major T.J., Dalbeth N., Stahl E.A., Merriman T.R. (2018). An update on the genetics of hyperuricaemia and gout. Nat. Rev. Rheumatol..

[110] El Ridi R., Tallima H. (2017). Physiological functions and pathogenic potential of uric acid: A review. J. Adv. Res..

[111] Casanova A.G., Morales A.I., Vicente-Vicente L., López-Hernández F.J. (2024). Effect of uric acid reduction on chronic kidney disease. Systematic review and meta-analysis. Front. Pharmacol..

[112] Su H.Y., Yang C., Liang D., Liu H.F. (2020). Research advances in the mechanisms of hyperuricemia-induced renal injury. BioMed Res. Int..

[113] Fu W., Liu Z., Wang Y., Li X., Yu X., Li Y., Yu Z., Qiu Y., Mei Z., Xu L. (2024). Baicalin inhibits monosodium urate crystal-induced pyroptosis in renal tubular epithelial cell line through Panx-1/P2X7 pathways: Molecular docking, molecular dynamics, and in vitro experiments. Chem. Biol. Drug Des..

[114] Liu Y.J. (2020). Study on the effect and mechanism of baicalin against hyperuricemia nephropathy. Master’s Thesis.

[115] Liu Z., Xiang H., Deng Q., Fu W., Li Y., Yu Z., Qiu Y., Mei Z., Xu L. (2023). Baicalin and baicalein attenuate hyperuricemic nephropathy via inhibiting PI3K/AKT/NF-κB signalling pathway. Nephrology.

[116] Xiang H., Lei H., Liu Z., Liu Y., Li Y., Qiu Y., Xu L. (2021). Network pharmacology and molecular docking analysis on molecular targets: Mechanisms of baicalin and baicalein against hyperuricemic nephropathy. Toxicol. Appl. Pharmacol..

[117] Meng X., Mao Z., Li X., Zhong D., Li M., Jia Y., Wei J., Yang B., Zhou H. (2017). Baicalein decreases uric acid and prevents hyperuricemic nephropathy in mice. Oncotarget.

[118] Chen Y., Zhao Z., Li Y., Yang Y., Li L., Jiang Y., Lin C., Cao Y., Zhou P., Tian Y. (2021). Baicalein alleviates hyperuricemia by promoting uric acid excretion and inhibiting xanthine oxidase. Phytomedicine.

[119] Wei X., Hou Y., Long M., Jiang L., Du Y. (2023). Advances in energy metabolism in renal fibrosis. Life Sci..

[120] Zhao D., Zhu X., Jiang L., Huang X., Zhang Y., Wei X., Zhao X., Du Y. (2021). Advances in understanding the role of adiponectin in renal fibrosis. Nephrology.

[121] Sears S.M., Sharp C.N., Krueger A., Oropilla G.B., Saforo D., Doll M.A., Megyesi J., Beverly L.J., Siskind L.J. (2020). C57BL/6 mice require a higher dose of cisplatin to induce renal fibrosis and CCL2 correlates with cisplatin-induced kidney injury. Am. J. Physiol. Ren. Physiol..

[122] Zhou S., Yin X., Yuan J., Liang Z., Song J., Li Y., Peng C., Hylands P.J., Zhao Z., Xu Q. (2022). Antifibrotic activities of Scutellariae Radix extracts and flavonoids: Comparative proteomics reveals distinct and shared mechanisms. Phytomedicine.

[123] Fang J., Wang W., Sun S., Wang Y., Li Q., Lu X., Qiu M., Zhang Y. (2016). Metabolomics study of renal fibrosis and intervention effects of total aglycone extracts of *Scutellaria baicalensis* in unilateral ureteral obstruction rats. J. Ethnopharmacol..

[124] Hu Q., Gao L., Peng B., Liu X. (2017). Baicalin and baicalein attenuate renal fibrosis in vitro via inhibition of the TGF-β1 signaling pathway. Exp. Ther. Med..

[125] Wang W., Zhou P.H., Xu C.G., Zhou X.J., Hu W., Zhang J. (2016). Baicalein ameliorates renal interstitial fibrosis by inducing myofibroblast apoptosis in vivo and in vitro. BJU Int..

[126] Wang W., Zhou P.H., Xu C.G., Zhou X.J., Hu W., Zhang J. (2015). Baicalein attenuates renal fibrosis by inhibiting inflammation via down-regulating NF-κB and MAPK signal pathways. J. Mol. Histol..

[127] Liang G.Q., Mu W., Jiang C.B. (2024). Baicalein improves renal interstitial fibrosis by inhibiting the ferroptosis in vivo and in vitro. Heliyon.

[128] Miao S.H. (2017). Effects of baicalein on endoplasmic reticulum stresspathway in rats with renal interstitial fibrosis. Master’s Thesis.

[129] Wang H., Jiang Q., Zhang L. (2022). Baicalin protects against renal interstitial fibrosis in mice by inhibiting the TGF-β/Smad signalling pathway. Pharm. Biol..

[130] Zheng L., Zhang C., Li L., Hu C., Hu M., Sidikejiang N., Wang X., Lin M., Rong R. (2017). Baicalin ameliorates renal fibrosis via inhibition of transforming growth factor β1 production and downstream signal transduction. Mol. Med. Rep..

[131] Liu H.X., Guo M.H., Liu Y., Zhu Y.J., Liu X.D., Xu Q.Y., Tan J. (2011). Effect of baicalin on human renal tubular epithelial cell transdifferentiation induced by TGF-β1 in vitro. Chin. J. Integr. Tradit. West. Nephrol..

[132] Tan Y.J., Zhu C.L., Mao H.X. (2016). Therapeutic effect of baicalin in treatment of renal interstitial fibrosis in rats with unliateral ureteral obstruction and related mechanisms. Chin. J. Contemp. Pediatr..

[133] Su N., Li F., Chen J.Y., Chen Z.X., Zhou L.Q., Luo R.J. (2009). Effect of baicalin on plasma and renal AngⅡ in diabetic nephropathy rats. Tradit. Chin. Drug Res. Clin. Pharmacol..

[134] Zhang S., Xu L., Liang R., Yang C., Wang P. (2020). Baicalin suppresses renal fibrosis through microRNA-124/TLR4/NF-κB axis in streptozotocin-induced diabetic nephropathy mice and high glucose-treated human proximal tubule epithelial cells. J. Physiol. Biochem..

[135] Hu H., Li W., Hao Y., Peng Z., Zou Z., Liang W. (2024). Baicalin ameliorates renal fibrosis by upregulating CPT1α-mediated fatty acid oxidation in diabetic kidney disease. Phytomedicine.

[136] Cai Y., Ma W., Xiao Y., Wu B., Li X., Liu F., Qiu J., Zhang G. (2017). High doses of baicalin induces kidney injury and fibrosis through regulating TGF-β/Smad signaling pathway. Toxicol. Appl. Pharmacol..

[137] Li F. (2011). The role of baicalin bai against glomerulosclerosis by regulating CTGF. Master’s Thesis.

[138] Zheng X.P., Feng J., Song C.Y., Wang F.F., Zhang H.M., Sui H.Y., Xin H., Chen G. (2020). Effect of baicalin on renal fibrosis in rats with diabetic nephropathy by TGF-β1. J. Med. Res. Combat. Trauma. Care.

[139] Liu Q.P., Shao X., Li Y.T., Liu M., Tan P., Wang Y. (2024). Baicalin improves renal fibrosis in rats with chronic kidney disease by regulating the galectin-3/Akt/GSK-3B/Snail signaling pathway. Hebei Med. J..

[140] Zhang X.T., Wang G., Ye L.F., Pu Y., Li R.T., Liang J., Wang L., Lee K.K.H., Yang X. (2020). Baicalin reversal of DNA hypermethylation-associated Klotho suppression ameliorates renal injury in type 1 diabetic mouse model. Cell Cycle.

[141] Nam J.E., Jo S.Y., Ahn C.W., Kim Y.S. (2020). Baicalin attenuates fibrogenic process in human renal proximal tubular cells (HK-2) exposed to diabetic milieu. Life Sci..

[142] Ke Y.Q., Hao J.P., Lu L., Chen X.L., Fu Y.Q. (2022). Study on the mechanism of wogonoside protecting against renal injury and fibrosis in diabetic rats. J. Guangzhou Univ. Tradit. Chin. Med..

[143] Meng X.M., Ren G.L., Gao L., Li H.D., Wu W.F., Li X.F., Xu T., Wang X.F., Ma T.T., Li Z. (2016). Anti-fibrotic effect of wogonin in renal tubular epithelial cells via Smad3-dependent mechanisms. Eur. J. Pharmacol..

[144] Zhang X.D., Ren G.L., Chen H.H., Chen Y., Zhu J. (2023). Effect of baicalin modulation of TLR4/MAPK/NF-KB signaling pathway on renal fibrosis in rats with diabetic nephropathy. Chin. Pharmacol. Bull..

[145] Kuo H.L., Chuang H.L., Chen C.M., Chen Y.Y., Chen Y.S., Lin S.C., Weng P.Y., Liu T.C., Wang P.Y., Huang C.F. (2024). Wogonin ameliorates ER stress-associated inflammatory response, apoptotic death and renal fibrosis in a unilateral ureteral obstruction mouse model. Eur. J. Pharmacol..

[146] Guo J., Mei Z.W., Wang X.J., Li Q., Qin J. (2023). Molecular docking and network pharmacological analysis of *Scutellaria baicalensis* against renal cell carcinoma. Eur. Rev. Med. Pharmacol. Sci..

[147] Wang Y. (2021). The role of wogonin in the malignant behavior and resistance to targeted drug of renal cell carcinoma. Ph.D. Thesis.

[148] Wang Y., Chen S., Sun S., Liu G., Chen L., Xia Y., Cui J., Wang W., Jiang X., Zhang L. (2020). Wogonin induces apoptosis and reverses sunitinib resistance of renal cell carcinoma cells via inhibiting CDK4-RB pathway. Front. Pharmacol..

